# Young Filipino breast cancer patients have worse survival outcomes

**DOI:** 10.3332/ecancer.2023.1639

**Published:** 2023-11-23

**Authors:** Ralph Victor Yap, Deanne Lou Marquez, Frances Marion De La Serna

**Affiliations:** 1 Department of Surgery, Cebu Doctors’ University Hospital, Osmeña Blvd, Cebu City, 6000, Philippines; 2 Unified Minimally Invasive Surgery Training, Quezon City, Manila, 1112, Philippines; a https://orcid.org/0000-0003-0083-696X; b https://orcid.org/0009-0003-4203-8461; c https://orcid.org/0000-0001-9632-5544

**Keywords:** breast cancer, young, survival, outcomes, Philippines

## Abstract

**Background:**

In the 2020 GLOBOCAN report, breast cancer is the 3rd most common cause of cancer-related mortality in the Philippines. The incidence of breast cancer in the young (≤40 years) was reported to be higher in the Philippines compared to other Asian countries. Several studies have consistently demonstrated poor survival outcomes in this age group due to its aggressiveness and unique tumour biology. However, data on survival outcomes of young Filipino breast cancer patients remains unknown in the Philippines.

**Methods:**

A retrospective study was performed involving patients with stage I–III breast cancer who underwent definitive surgery from January 2010 to December 2015 at a single-tertiary institution. Patients were grouped according to age (≤40 and >40 years old). Their clinicopathological characteristics, treatment profile and 5-year survival outcomes were analyzed.

**Results:**

A total of 524 Filipino patients (15.1% aged ≤40 years) were included. Younger patients were diagnosed at a higher stage and pathologic grade. A negative hormone receptor, high Ki67 status, and triple negative breast cancer (TNBC) subtypes were also more common among younger patients. The overall breast-conserving surgery rate was low at 8.9%. The use of adjuvant chemoradiotherapy was more common and both 5-year overall survival (OS) and disease-free survival (DFS) were lower (61.1% versus 77.1% and 31.1% versus 66.8%, respectively) in the ≤40-year-old group. In the multivariate analysis, age group, tumour size, and nodal status were significant predictors for DFS. However, only tumour size was significant for OS.

**Conclusion:**

Young Filipino breast cancer patients have demonstrated unique pathologic characteristics with associated lower survival outcomes similar to the published literature. Increasing awareness of cancer screening practices among young women, provision of equitable access to healthcare, and prompt management of breast cancer in the young are crucial.

## Background

According to the GLOBOCAN 2020 report, female breast cancer has become the most commonly diagnosed cancer worldwide with an estimated 2.3 million new cases [1]. In the Philippines, it is the 3rd most common cause of cancer-related mortality [2]. Additionally, it was reported that 1 out of 13 Filipino women is expected to develop breast cancer in their lifetime [3]. The incidence of breast cancer continues to increase with age while the median age at the time of breast cancer diagnosis is 61 years [4].

Interestingly, the incidence of breast cancer in young women has been on the rise, with breast cancer as the second leading cause of cancer-related mortality in women aged <39 years. Although the age cut-off for breast cancer in the young is inconsistently defined in previous literature (<45, <40, <35 years), the European School of Oncology – European Society for Medical Oncology 3rd international consensus guidelines in 2017 defined ‘young women’ as less than 40 years of age at the time of pathologic diagnosis [5].

Data analysed from the California Cancer Registry (1998–2004) have shown that migrant Filipino women were being diagnosed at a statistically younger age compared to Caucasians and other Asian women [6]. In Asia, up to 13% of women diagnosed with breast cancer are aged <40 years. The incidence of breast cancer among this age group is particularly higher in the Philippines (22.1 per 100,000 women) compared with Osaka (11.8), Hanoi (13.8), 14.7 (Shanghai), Bangkok (15.2) and Hong Kong (19.3) [7, 8].

Despite worldwide trends in improvements in early detection and treatment access of breast cancer, the recurrence and mortality rates among young women with breast cancer remain poor. The survival disparity in young breast cancer patients could be attributed to its aggressiveness and unique biological features compared to older women. Breast cancer in the young tends to present at an advanced stage, with higher rates of triple-negative or human epidermal growth factor 2 (HER2)-overexpression profiles, and with lower efficacy to hormonal therapy [6, 9].

Moreover, breast cancer with breast cancer gene (BRCA) mutations was more common among Filipino patients aged <45 years and associated with worse overall survival (OS) [10, 11]. Optimizing the care and outcomes for this population would require a multidisciplinary team while tailoring therapies that would also address specific issues on fertility preservation, pregnancy, survivorship and psychosocial support [12].

To the best of our knowledge, there have been no publications on the clinicopathologic and survival outcomes of young Filipino women with breast cancer. Thus, this study aims to compare the clinical and treatment profiles, OS, and disease-free survival (DFS) between the young (≤40 years) and older Filipino women with breast cancer treated at a private, tertiary hospital in the Philippines.

## Methods

This was a retrospective study on all patients with pathologically confirmed primary stages I–III breast cancer aged 18 years old and above who underwent definitive surgery from January 01, 2010, to December 31, 2015, at Cebu Doctors’ University Hospital. This study was conducted in accordance with the Declaration of Helsinki, and the institution’s research ethics committee approved the study (protocol code 2-2019-034). The hospital’s medical records and outpatient clinic data were utilized.

The variables collected from the patient’s medical records included clinicopathologic characteristics – age (at the time of pathological diagnosis); histology, tumour size, nodal status, stage, tumour grade, lymphovascular invasion, hormonal and HER2/neu receptor status, Ki67 value and molecular subtype) from the records of the Department of Pathology. The operative records were reviewed to extract data on the surgical therapy to the breast (lumpectomy/breast-conserving surgery (BCS) or total mastectomy, and the method of axillary staging such as sentinel lymph node biopsy (SLNB) or axillary lymph node dissection (ALND). Neoadjuvant and adjuvant therapies (radiation therapy (RT) and systemic/chemotherapy) were reviewed using the patient’s medical charts, outpatient records, and/or records from the institution’s medical and radiation oncology departments. The hospital medical records and the prospectively maintained outpatient clinic records of the patients were reviewed to determine recurrence and survival status. DFS was defined as the time interval between the date of definitive surgery and the development of local/regional recurrence and/or development of distant metastasis. OS was defined as the time interval between the date of definitive surgery and mortality regardless of cause. The last follow-up date was January 01, 2021. Patients with a previous history of or synchronous malignancy other than the breast, whose records do not contain follow-up data, and male breast cancer were excluded from the study.

All collected data was encoded in Microsoft Excel (Microsoft Corp., Redmond, WA, USA). Patients were grouped according to age (≤40 and >40 years old). Values were expressed as frequencies (percentages) and analysed using chi-square or Fisher’s exact test whenever appropriate. The primary and secondary endpoints of this study were to assess the 5-year OS and DFS between the 2 groups, respectively using the Log Rank (Mantel-Cox) test and Kaplan-Meier curve plot. Multivariate analyses for OS and DFS were done using Cox regression analysis. The following variables were included in the model: age groups (age <40 and age ≥40), pathologic *T* stage, *N* stage, final stage, grade, LVI, oestrogen receptor (ER), progesterone receptor (PR) and Ki67 status. We evaluated the hazard ratio and its 95% confidence interval for each variable. A *p*-value of <0.05 was considered significant in all analyses.

All statistical analysis was performed using GraphPad Prism version 8.4.2 (GraphPad Software, San Diego, CA, USA) and IBM SPSS Statistics version 26 (IBM Corp., Armonk, NY, USA) for Windows.

## Results

### Clinicopathological characteristics

A total of 524 female Filipino breast cancer patients were included in the study. Of these, 81 (15.5%) patients were aged ≤40 years old (mean 34.9 ± 5.1) while 443 (84.5%) were aged >40 years old (mean 56.7 ± 10.6). Overall, invasive ductal carcinoma (IDC) was the most common histologic type (75.4% versus 56.2%, *p* = 0.0035). There were no significant differences between the 2 groups in terms of tumour size, nodal status and LVI. Patients aged ≤40 years old were diagnosed at a higher stage and pathologic grade, with higher negative rates for ER (48.1% versus 29.1%) and PR (59.3% versus 45.8%) receptor status, and Ki67 high status (45.7% versus 26.2%). A lower luminal A/B (29.6% versus 48.5%) and higher triple-negative (TNBC) (16% versus 7.2%) molecular subtypes were found among patients ≤40 years old (Table 1).

### Treatment profile

The total mastectomy rates between the ≤40 and >40-year-old groups were 88.9% and 91.4%, respectively. Overall, the BCS rate was only 8.9%. More patients in the ≤40-year-old group received adjuvant chemotherapy (45.7% versus 33.2%) and RT (50.6% versus 31.6%). There were no significant differences between the 2 groups in terms of the method of axillary staging, neoadjuvant chemotherapy and adjuvant hormonal and anti-HER2/neu therapies (Table 2).

### Survival outcomes

**Figure 1.**
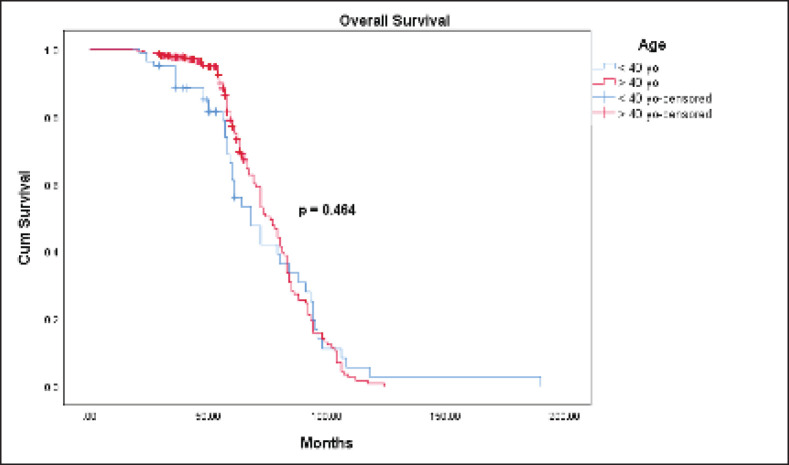
OS between the ≤40 and >40-year-old groups with breast cancer.

**Figure 2.**
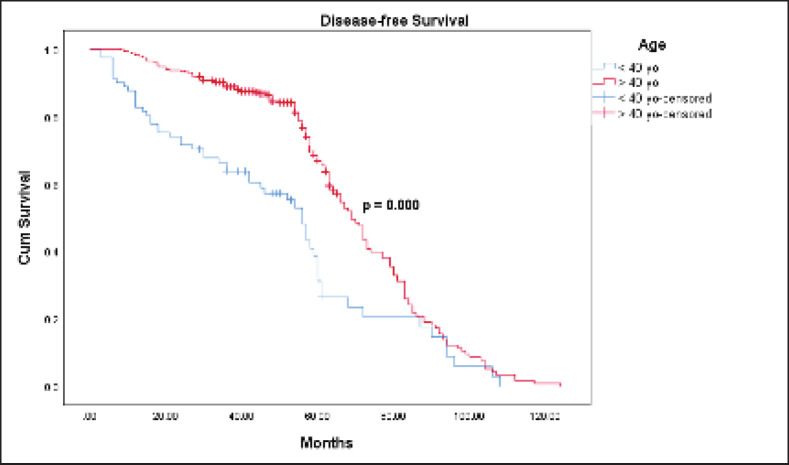
DFS between the ≤40 and >40-year-old groups with breast cancer.

The Kaplan-Meier estimates indicated that the 5-year OS rate for the ≤40-year-old group was 61.1% and for the >40-year-old group was 77.1%. However, the Log Rank test indicated there was no statistically significant difference (*p* = 0.464) between the two survival rates (Figure 1). The mean OS time for the ≤40-year-old group was 74 months and for the >40-year-old group was 76.2 months. The 5-year DFS rate for the ≤40-year-old group was significantly lower (31.1% versus 66.8%, *p* = 0.000) than the >40-year-old group (Figure 2). The mean DFS time for the ≤40-year-old group was 50.5 months and for the >40-year-old group was 69 months. Using the Cox regression model, the multivariate analysis for DFS showed that younger age (≤40 years), higher tumour size, and nodal status were significant in the model controlling for all other variables. However, only tumour size was significant when analysing for OS. Patients in the ≤40-year-old group had 1.9 times the risk of their older counterparts in developing disease recurrence (95% CI (1.391–2.694), *p* = 0.000) (Table 3).

## Discussion

Our study demonstrated that young Filipino women (≤40-year-old) with breast cancer were diagnosed with a higher pathologic grade and stage, had more IDC pathology, hormonal receptor negativity, high Ki67 status and TNBC molecular subtypes, and had more adjuvant chemo- and radiotherapy when compared with older patients. Moreover, young women with breast cancer had lower 5-year OS and DFS.

We observed in our study that young breast cancer patients had significantly more IDC and less invasive lobular carcinoma (ILC) pathologies compared to the older cohort. A similar pathologic finding was also noted in a Jordanian cohort (970 patients). However, the young breast cancer patients in their study had significantly better OS and comparable DFS [13]. Previous reports from India (507 patients) and China (1,968 patients) did not find significant differences in histologic types between young (≤40-year-old) and non-young breast cancer patients [14, 15]. In a large cohort of 17,481 breast cancer patients in Sweden, ILC was found to be significantly associated with older age, ER positivity and well-differentiated tumours. Additionally, ILC was noted with improved survival during the first 5 years [16].

Young women with breast cancer have unique pathologic characteristics with an aggressive phenotype compared to older patients. Although our study did not demonstrate statistically significant differences in terms of pathologic tumour size, nodal status, and LVI, previous reports have shown that young breast cancer patients tend to present with T3/T4 tumour size (28.2% – 63.1% versus 13.8% – 15.2%), nodal positivity (73.2% – 86.7% versus 55.6% – 75.5%) and positive LVI (39.3% – 48.6% versus 27.7% – 39.4%) [13, 14, 17, 18]. Whether this is secondary to a small sample size and/or racial/geographical difference remains to be investigated. Other findings in our study (more pathologic grade 3, stage 3, higher Ki67 status and more hormonal receptor (ER/PR) negative, and TNBC subtypes) were consistent with previously published data from Jordan, India, Argentina and Croatia [13, 14, 18, 19]. All these variables are considered prognostic factors that negatively impact OS and DFS.

Additionally, younger patients with breast cancer were more likely to present with a positive HER2/neu receptor status, a marker for aggressive tumour phenotype, poor prognosis and targeted/chemotherapy [17, 18]. However, we could not confirm this in our study due to the disproportionate number of patients with unknown or unconfirmed HER2/neu status. In a 2015 local survey, more than 90% of medical oncologists and surgeons would request HER2/neu testing for their breast cancer patients. However, common barriers such as the unavailability of biomarker testing, patients’ refusal and limited finances remain a hindrance in pursuing this test in the Philippines [20, 21]. Secondly, only 1.3% of our patients had documented adjuvant anti-HER2/neu therapy. In a cost-utility analysis in the Philippines, adjuvant trastuzumab therapy (all cycles) for HER2/neu positive early breast cancer was not cost-effective (in addition to chemotherapy) and remains unaffordable at its 2017 government-negotiated price of PHP 619,667 (USD 11,161) [22].

No significant difference was found in terms of surgical therapy for the breast and method of axillary staging between the two age groups in our study. Only less than 12% of the young breast cancer patients had BCS, a rate similar to what we previously reported among early-stage breast cancer patients aged ≥18 years old in our institution. Multiple factors such as co-morbidities, education level, socio-economic and marital status, and mode of diagnosis may influence the decision to pursue BCS [23]. We also observed in our local practice that patients opt for total mastectomy because of the fear of having a second surgery for recurrence (for leaving breast tissue behind), and the additional financial burden of having to undergo adjuvant breast RT after lumpectomy. Similar to a recent survey among 383 Lebanese women with a median age of 32 years, ‘concern about residual cancer and/or cancer recurrence’, ‘cost of surgery and follow-up and the absence of health coverage’, and ‘side effects of radiotherapy’ were one of the main reasons for choosing mastectomy over BCS [24]. In a survival analysis of 15,611 breast cancer patients aged ≤40 years old utilizing the National Cancer Database (2006–2016), the BCS rate was 60.9% with an equivalent 5-year OS to mastectomy with or without RT [25].

In our study, the rates of adjuvant chemotherapy and radiation therapy were higher among women in the ≤40-year-old group. The unique pathologic characteristics as previously mentioned justify its increased use in this age group. While selecting chemotherapeutic regimens remains the same regardless of age, the comorbidities associated with older women may preclude the use or require dose modifications of systemic therapies [26]. Recommendations for adjuvant RT are similar to those in older women. However, the risk of local recurrence is higher among young women not only after BCS but also after mastectomy thus they are more likely to benefit from RT than their older counterparts [27].

### Survival outcomes from neighbouring Southeast Asian countries

Our study demonstrated that the 5-year OS (61.1% versus 77.1%) and DFS (31.1% versus 66.8%) were lower in the ≤40-year-old group. We reviewed the literature on the survival outcomes of young breast cancer patients from other southeast Asian countries.

In Indonesia, a comparative study between young (≤40 years old) and elderly (≥60 years old) breast cancer patients showed that the former group had more positive lymph nodes, HER2/neu receptor positivity, adjuvant chemotherapy and tamoxifen use. Secondly, 5-year mortality, recurrence, and metastasis rates were also higher (30.4% versus 20%, 7.95 versus 5.7%, and 25% versus 22.8%, respectively) in the same group as well [28]. A more recent study from Indonesia analysing 115 young breast cancer patients showed a shorter progression-free survival in those with T4 tumours (16 versus 35 months), positive lymph nodes (24 versus 42 months), hormone receptor-positive (29 versus 24 months) and TNBC subtype (16 versus 38 months). Within 5 years of follow-up, 6.9% and 24.3% developed locoregional recurrence and distant metastasis, respectively [29]. In a cohort of stage III Indonesian breast cancer patients, those aged ≤40 years had a lower 3-year OS of 47% (versus 78%, *p* 0.010) [30].

A survival analysis of 868 breast cancer patients in Malaysia (comprised of 58% Malays, 25% Chinese and 17% Indians) showed a lower 5-year OS at 57.4% in the <40-year-old group [31]. A multivariable Cox regression analysis in 290 Thai women with breast cancer (9% were aged 35 years or younger) who underwent BCS showed that young age was a risk factor for locoregional (HR 4.1, *p* 0.01), and distant (HR 1.7, *p* 0.001) recurrences [32].

A 2005 study conducted in Singapore comparing 106 young breast cancer patients (higher tumour size, pathologic grade and more nodal positive) to 737 older counterparts showed a 5-year OS of 86.4% and 81.7%, respectively. However, this was not statistically significant [33]. In 2009, data from 10,287 Singaporean women with breast cancer with a median follow-up of 7.7 years was analysed. Those who were primarily diagnosed at the age of 35 to 54 years had a statistically significant 34% lower risk of mortality compared to patients aged <35 years [34]. A more recent (2018) Singaporean study comparing the survival outcomes between young (447) and old (2,045) breast cancer patients treated with BCS showed similar 5-year OS rates (94.1% versus 96.1%, respectively). However, the 5-year breast cancer-specific survival was significantly lower in the young group (96.7% versus 98.3%). Additionally, younger patients were more likely to have breast cancer recurrence (HR 1.92, *p* < 0.001) with 5-year local recurrence rates of 5.2% versus 3%, respectively [35].

The lower survival outcomes among young Filipino breast cancer patients were similar to other Southeast Asian countries. The lack of effective screening strategies for average-risk young women may result in the late stage of breast cancer presentation. A cross-sectional study involving 994 Filipino women (33% were aged 20–39 years) not diagnosed with breast cancer showed less reported knowledge about clinical breast exams (CBE) or mammograms. Adherence to CBE and monthly breast self-exams were only 15% and 25%, respectively. Only 8% of the participants had a prior mammogram which was generally done for diagnostic rather than screening purposes [3].

The high out-of-pocket healthcare costs, lack of organized national screening programs, centralization and/or unequal distribution of health resources/infrastructure and providers across the country and socioeconomic and cultural barriers preclude access to both breast cancer screening and timely surgical management [36, 37]. Lastly, specific challenges in the care of young breast cancer patients such as fertility preservation/future pregnancy, bone health maintenance, inherited breast cancer syndromes, and associated psychosocial issues should be included and discussed during multidisciplinary treatment planning [38, 39].

Our study is limited by its retrospective design, data incompleteness of hormonal receptor, HER2/neu, and Ki67 status, and a small study sample based solely on a single private tertiary institution, and outcomes may not be generalizable for the entire region/country. Secondly, a standardized and stringent research database/registry for breast cancer patients to optimize outcomes is still lacking in our setting.

## Conclusion

We describe for the first time, survival outcomes of young (≤40 years) breast cancer patients in the Philippines. The unique pathologic characteristics and associated low OS and DFS in this group were consistent with published literature. Increasing awareness about breast cancer screening practices among young women, providing equitable/affordable access to healthcare, and prompt management of breast cancer in the young are imperative.

## Disclosure

This paper was presented virtually as a poster presentation (by DLM) during the 11th Global Breast Cancer Conference held at Grand Walkerhill Hotel, Seoul last April 28–30, 2022.

## Conflicts of interest

The authors declare that they have no conflicts of interest.

## Funding

The authors declare that they have not received any funding for the execution of this research/article.

## Author contributions

RVY, DLM and FMD conceptualized the study design. DLM collected the data. RVY conducted the data analysis. RVY and DLM interpreted the results and wrote the manuscript. FMD gave critical comments and revisions to the manuscript. All authors approved the final version of the manuscript before submission.

## Figures and Tables

**Table 1.** Clinicopathological characteristics between the ≤40 and >40-year-old groups with breast cancer.

	≤40 years old *N* = 81	>40 years old *N* = 443	*p*-value
Histology			0.0035*
Invasive ductal	61 (75.4)	249 (56.2)	
Invasive lobular	10 (12.3)	124 (28)	
Mixed/Others	10 (12.3)	70 (15.8)	
Tumour size^a^			0.2869
pT1	15 (18.5)	118 (26.3)	
pT2	44 (54.3)	239 (54)	
pT3	11 (13.6)	43 (9.7)	
pT4	11 (13.6)	43 (9.7)	
Nodal status^a^			0.4954
pN0	38 (46.9)	235 (53)	
pN1	25 (30.9)	111 (25)	
pN2	13 (16)	58 (13.1)	
pN3	5 (6.2)	39 (8.8)	
Stage^a^			0.0130*
1/2	50 (61.7)	336 (75.8)	
3	31 (38.3)	107 (24.2)	
Grade			0.0034*
1	20 (24.7)	173 (39)	
2	34 (42)	189 (42.7)	
3	27 (33.3)	81 (18.3)	
Positive LVI	28 (34.6)	129 (29.1)	0.3562
ER			0.0004*
Positive	42 (51.9)	279 (63)	
Negative	39 (48.1)	129 (29.1)	
Unknown	0	35 (7.9)	
PR			0.0089*
Positive	33 (40.7)	205 (46.3)	
Negative	48 (59.3)	203 (45.8)	
Unknown	0	35 (7.9)	
HER2/neu status			0.0098*
Positive	15 (18.5)	88 (19.9)	
Negative	56 (69.1)	235 (53)	
Unknown	10 (12.4)	120 (27.1)	
Ki67 status			0.0006*
Low (≤14%)	11 (13.6)	124 (28.0)	
High (>14%)	37 (45.7)	116 (26.2)	
Unknown	33 (40.7)	203 (45.8)	
Molecular subtype			0.0098*
Luminal A	15 (18.5)	139 (31.4)	
Luminal B	9 (11.1)	76 (17.1)	
HER2/neu enriched	14 (17.3)	69 (15.6)	
Triple negative	13 (16)	32 (7.2)	
Unknown	30 (37)	127 (28.7)	

* Statistically significant;

a Based on the 8^th^ AJCC Cancer Staging Manual; HER2/neu, Human epidermal growth factor receptor 2; LVI, Lymphovascular invasion

**Table 2.** Treatment profile between the ≤40 and >40-year-old groups with breast cancer.

	≤40 years old *N* = 81	>40 years old *N* = 443	*p*-value
Treatment of breast			0.5243
Total mastectomy	72 (88.9)	405 (91.4)	
Lumpectomy/BCS	9 (11.1)	38 (8.6)	
Treatment of axilla			0.2494
SLNB	14 (17.3)	106 (23.9)	
ALND	67 (82.7)	337 (76.1)	
Neoadjuvant chemotherapy			0.8822
Yes	16 (19.8)	94 (21.2)	
No	65 (80.2)	349 (78.8)	
Adjuvant chemotherapy			0.0322
Yes	37 (45.7)	147 (33.2)	
No	44 (54.3)	296 (66.8)	
Adjuvant hormonal therapy			0.2747
Yes	31 (38.3)	200 (45.1)	
No	50 (61.7)	243 (54.9)	
Adjuvant anti-HER2/neu therapy			>0.9999
Yes	1 (1.2)	6 (1.4)	
No	80 (98.8)	437 (98.6)	
Adjuvant radiation therapy			0.0014*
Yes	41 (50.6)	140 (31.6)	
No	40 (49.4)	303 (68.4)	

* Statistically significant; ALND, Axillary lymph node dissection; BCS, Breast-conserving surgery; HER2/neu, human epidermal growth factor receptor 2; SLNB, Sentinel lymph node biopsy

**Table 3.** Multivariate cox regression analysis for DFS and OS in the population.

Variable	DFS		OS	*p*-value
	HR (95% CI)	*p*-value	HR (95% CI)	
Age, years				
>40 years old	Reference		Reference	
≤40 years old	1.935 (1.391–2.694)	0.000*	1.047 (0.704–1.557)	0.819
Tumor size				
pT1	Reference	0.000*	Reference	0.000*
pT2	1.113 (0.767–1.614)	0.573	1.017 (0.681–1.518)	0.935
pT3	0.794 (0.404–1.561)	0.504	0.781 (0.351–1.742)	0.546
pT4	3.936 (2.032–7.622)	0.000*	4.235 (1.709–10.494)	0.002*
Nodal status				
pN0	Reference	0.012*	Reference	0.604
pN1	1.004 (0.682–1.480)	0.982	0.980 (0.638–1.505)	0.927
pN2	1.450 (0.884–2.380)	0.142	1.376 (0.707–2.680)	0.347
pN3	2.274 (1.339–3.862)	0.002*	1.469 (0.758–2.848)	0.254
Stage				
1/2	Reference		Reference	
3	1.601 (0.902–2.841)	0.108	1.118 (0.543–2.303)	0.762
Grade				
1	Reference	0.814	Reference	0.591
2	1.110 (0.790–1.559)	0.547	0.886 (0.612–1.284)	0.523
3	1.112 (0.739–1.674)	0.610	1.102 (0.689–1.764)	0.685
LVI				
Negative	Reference		Reference	
Positive	0.912 (0.640–1.300)	0.610	0.725 (0.472–1.116)	0.144
ER				
Positive	Reference	0.865	Reference	0.644
Negative	0.908 (0.622–1.326)	0.617	0.890 (0.597–1.327)	0.568
Unknown	1.086 (0.475–2.481)	0.845	1.600 (0.456–5.617)	0.463
PR				
Positive	Reference	0.143	Reference	0.073
Negative	1.320 (0.910–1.912)	0.143	1.440 (0.966–2.145)	0.073
Unknown	Not included		Not included	
Ki67 status				
Low (≤14%)	Reference	0.056	Reference	0.000*
High (>14%)	0.845 (0.573–1.248)	0.398	0.769 (0.515–1.148)	0.198
Unknown	0.590 (0.380–0.916)	0.019	0.265 (0.137–0.513)	0.000*

* Statistically significant; LVI, Lymphovascular invasion
